# SMARCB1-deficient sinonasal carcinoma in a pediatric patient: Imaging findings and differential diagnosis of a rare entity

**DOI:** 10.1016/j.radcr.2026.08.069

**Published:** 2026-09-16

**Authors:** Jonathan Yoon, Amanda Aguilera

**Affiliations:** a School of Medicine, Loma Linda University, Loma Linda, CA 92354, USA; b Department of Radiology, Loma Linda University Health, Loma Linda, CA 92354, USA

**Keywords:** SMARCB1, Sinonasal carcinoma, Pediatric, Skull base, CT, MRI, INI1, Case report

## Abstract

SMARCB1-deficient sinonasal carcinoma (SDSC) is a recently characterized and aggressive sinonasal malignancy described predominantly in adults, with only rare reports in patients under 18 years of age and limited radiologic characterization in young children. We present the case of an 8-year-old boy with new-onset vision loss found to have a large, heterogeneously enhancing central skull base mass demonstrating diffusion restriction, internal sheet-like calcification, extensive osseous destruction, and multicompartment skull base extension involving the cavernous sinuses, orbital apices, and internal carotid arteries. The imaging differential diagnosis included rhabdomyosarcoma, esthesioneuroblastoma, poorly differentiated chordoma, craniopharyngioma, germ cell tumor, and NUT carcinoma. Endoscopic biopsy demonstrated complete loss of INI1/SMARCB1 expression on immunohistochemistry, consistent with SMARCB1-deficient sinonasal carcinoma. Despite multimodal therapy including chemotherapy, radiation, immunotherapy, and subtotal resection, the tumor demonstrated progressive local and metastatic disease with subsequent vascular complications including internal carotid artery encasement and subsequent cerebral infarction. This case highlights the aggressive imaging appearance of SDSC in a pediatric patient and underscores the importance of including this entity in the differential diagnosis of destructive pediatric skull base masses to prompt appropriate immunohistochemical evaluation and ensure accurate pathologic classification.

## Introduction

Sinonasal malignancies are rare, accounting for approximately 5% of head and neck cancers, with an overall incidence of 0.83 per 100,000 individuals annually [1]. The incidence in pediatric patients is significantly lower, estimated at 0.036 per 100,000 per year [2]. Despite their rarity, sinonasal tumors demonstrate considerable variability in histology, clinical behavior, and prognosis. Imaging plays a central role in initial detection, lesion characterization and extent as well as guidance of biopsy planning.

SMARCB1-deficient sinonasal carcinoma (SDSC) is a recently recognized and aggressive malignancy characterized by loss of SMARCB1 (INI1) expression on immunohistochemistry. SMARCB1 is a core subunit of the SWI/SNF chromatin remodeling complex that regulates nucleosome positioning and gene expression and functions as a tumor suppressor [3]. Loss of SMARCB1 expression has been well described in other malignancies, including atypical teratoid/rhabdoid tumor and malignant rhabdoid tumor, but its identification in primary sinonasal carcinoma was first reported in 2014 [4]. Since that time, fewer than 200 cases have been described, predominantly in middle-aged adults, with a reported median age in the fifth to sixth decade of life [5].

Imaging findings of SDSC in adults typically reflect an aggressive high-grade malignancy, often demonstrating heterogeneous enhancement, diffusion restriction, osseous destruction, and early skull base invasion [5]. Calcifications have been reported in approximately half of adult cases, with a subset demonstrating a ``hair-on-end'' periosteal pattern suggestive of aggressive periosteal reaction [5]. SDSC typically presents with locoregionally advanced disease at diagnosis and is often associated with poor prognosis compared to tumors with retained SMARCB1 expression [6,7]. To date, reports of SDSC in pediatric patients are exceedingly limited, and detailed radiologic descriptions in this population are lacking.

We report a case of SMARCB1-deficient sinonasal carcinoma in an 8-year-old child, with emphasis on multimodality imaging findings, radiologic differential diagnosis, and imaging features that may prompt early consideration of this entity in pediatric sinonasal and skull base masses.

## Case report

### Clinical presentation

An 8-year-old previously healthy male presented with new-onset vision loss in the right eye. Visual acuity screening was abnormal, and ophthalmologic evaluation identified a right afferent pupillary defect. Initial laboratory evaluation, including complete blood count with differential, comprehensive metabolic panel, syphilis total antibody testing, and QuantiFERON-TB Gold testing, was unremarkable.

### Initial imaging

**Fig. 1:**
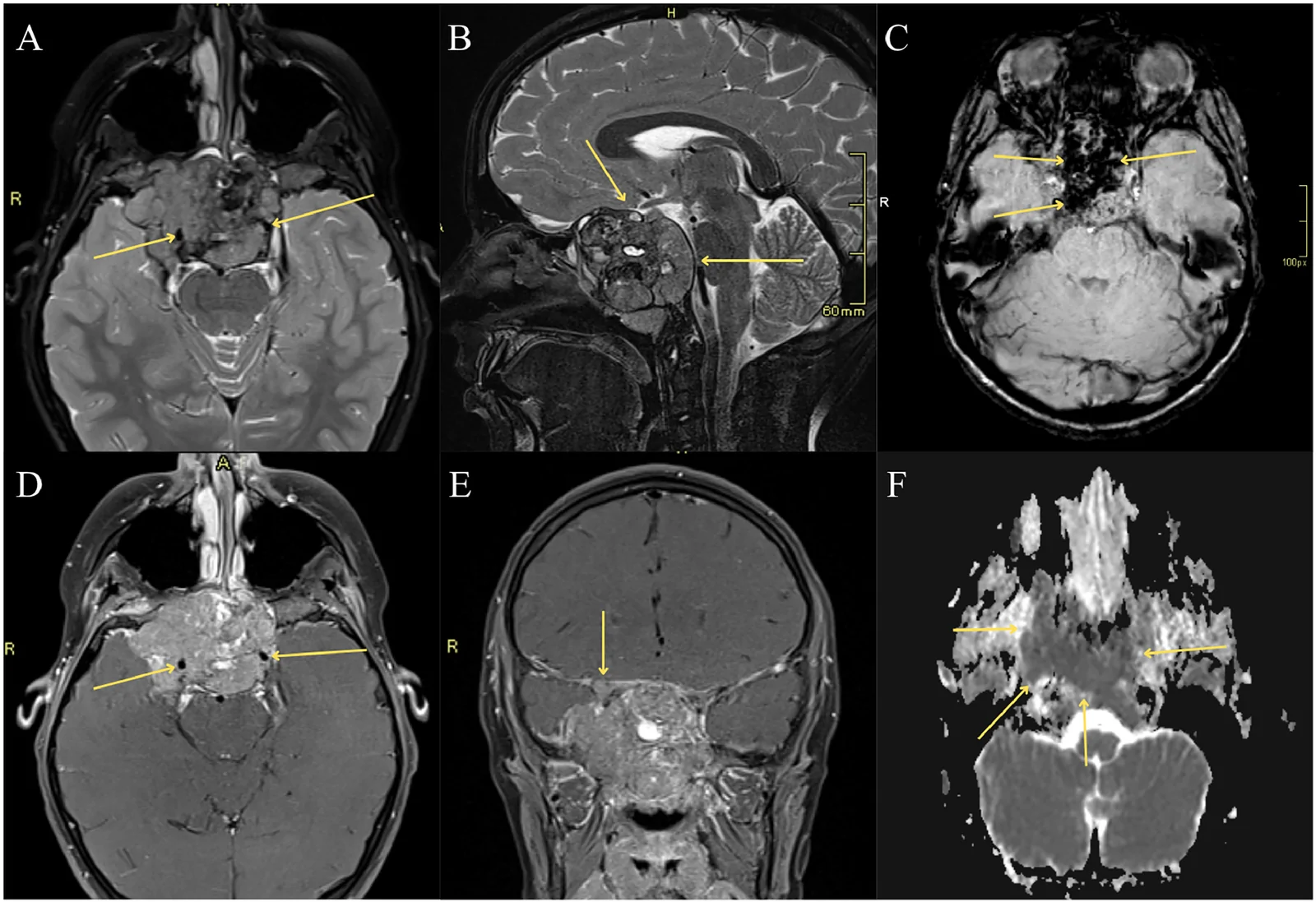
Multiplanar MRI of SMARCB1-deficient sinonasal carcinoma in an 8-year-old patient. (A) Axial T2-weighted image demonstrates a large, heterogeneous central skull base mass, eccentric to the right, with involvement of the sphenoid sinuses and encasement of the bilateral internal carotid artery flow voids (arrows). (B) Sagittal T2-weighted image demonstrates the extent of craniocaudal involvement of the central skull base with extension into the sellar and clival regions (arrows). (C) Axial susceptibility-weighted imaging (SWI) demonstrates multiple foci of susceptibility hypointensity within the lesion (arrows), corresponding to calcification and intralesional hemorrhage. (D) Axial contrast-enhanced T1-weighted image demonstrates heterogeneous enhancement with encasement of the bilateral internal carotid arteries (arrows) and extension toward the right pterygopalatine region. (E) Coronal contrast-enhanced T1-weighted image demonstrates the large heterogeneously enhancing central skull base mass with involvement of the right orbital apex (arrow). (F) Apparent diffusion coefficient (ADC) map demonstrates low ADC values within the lesion (arrows) with a mean value of 0.603 × 10⁻³ mm²/s, consistent with restricted diffusion in a hypercellular neoplasm.

Contrast-enhanced MRI of the brain, orbits, face, and neck demonstrated a large, heterogeneously enhancing central skull base mass measuring approximately 4.7 × 5.9 × 4.3 cm (anteroposterior × transverse × craniocaudal) (Fig. 1). The mass was centered in the central skull base with involvement of the bilateral sphenoid sinuses, bilateral greater sphenoid wings, and clivus, overall worse on the right. Multiple internal foci of T1 hyperintensity were present, likely reflecting hemorrhagic or proteinaceous components, with a few areas of internal T2 hypointense signal and focal susceptibility hypointensity on SWI sequences. The mass was predominantly solid with minimal internal cystic change. Diffusion-weighted imaging demonstrated moderate diffusion restriction consistent with hypercellularity.

Superiorly, the mass extended into the sellar and suprasellar regions with superior displacement of the pituitary gland, which appeared compressed along its inferior margin but otherwise preserved. There was encasement of the bilateral internal carotid arteries and mass effect on the optic chiasm. The right orbital apex was involved with narrowing of the right optic canal, corresponding to the patient's presenting visual symptoms; the left orbital apex was spared. Posteriorly, the mass expanded the clivus with extension into the prepontine cistern. Laterally, there was involvement of the bilateral middle cranial fossae with mild mass effect on the bilateral temporal lobes, right greater than left, and widening of the right foramen ovale. There was associated extension into the right masticator space, with slight bulging into the right pterygopalatine fossa. Anteriorly, the mass extended to involve the posterior aspect of the bilateral ethmoid air cells and anterior cranial fossae, right greater than left. Inferiorly, the mass extended into the posterior nasal cavity and nasopharynx bilaterally.

**Fig. 2:**
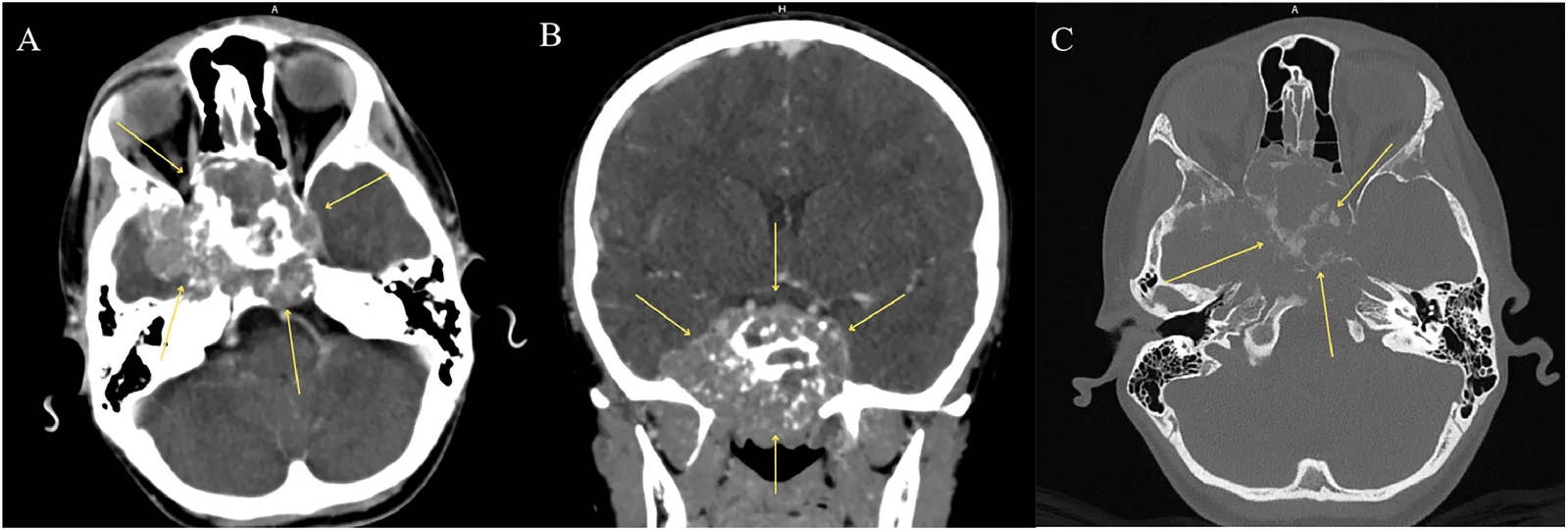
Contrast-enhanced CT of SMARCB1-deficient sinonasal carcinoma in an 8-year-old patient. (A) Axial and (B) coronal post-contrast soft tissue images demonstrate a lobulated, enhancing central skull base mass (arrows), eccentric to the right. Internal hyperattenuation within the lesion corresponds to areas of calcification. (C) Axial bone window image better demonstrates prominent sheet-like calcifications within the lesion (arrows).

Contrast-enhanced CT demonstrated osseous destruction of the central skull base by the heterogeneously enhancing destructive mass, with amorphous internal sheet-like calcifications corresponding to the areas of susceptibility hypointensity identified on MRI (Fig. 2). PET imaging revealed intense hypermetabolic activity within the mass with a maximum standardized uptake value of 26.9, consistent with a highly metabolically active malignancy.

### Imaging differential diagnosis

Based on patient age and imaging findings, the differential diagnosis for an aggressive pediatric central skull base mass included rhabdomyosarcoma, esthesioneuroblastoma, chordoma, craniopharyngioma, germ cell tumor, and NUT (Nuclear protein in Testis) carcinoma.

Rhabdomyosarcoma was considered the primary differential given the patient's age and the aggressive osseous destruction with diffusion restriction; however, the presence of internal calcification and the degree of multicompartment skull base invasion were atypical. Esthesioneuroblastoma was considered given the sinonasal origin and skull base involvement, though the extensive osseous destruction, diffusion restriction, and internal calcification pattern were unusual for this etiology.

Chordoma was considered given the clival involvement and central skull base epicenter. Classic conventional chordoma was considered less likely given the absence of the characteristic T2 hyperintensity typically attributed to physaliphorous cells and myxoid stroma; however, poorly differentiated chordoma, a variant that preferentially affects children and young adults, was a relevant consideration [8]. Poorly differentiated chordoma demonstrates loss of SMARCB1 expression and shares several aggressive imaging features with SDSC including osseous destruction, diffusion restriction, and absence of T2 hyperintensity, underscoring that immunohistochemical evaluation is essential for distinguishing between these entities [9].

Craniopharyngioma was considered given the suprasellar extension and presence of calcification; however, the true epicenter in the central skull base and sinonasal region, combined with the degree of osseous destruction and diffusion restriction, argued against this diagnosis. Germ cell tumor was also considered given the patient's age and midline location, though the absence of characteristic fat-containing components and the pattern of osseous destruction made this diagnosis less likely. NUT carcinoma was also a consideration given the patient's young age and the midline sinonasal location, as this entity shares many aggressive imaging features with SDSC and can only be reliably distinguished on immunohistochemical and molecular testing [10].

Given the aggressive imaging characteristics, extensive multicompartment skull base invasion, internal calcification, and diffusion restriction, a high-grade malignancy was favored, and tissue diagnosis was pursued. Differential considerations are summarized in Table 1.

**Table 1:** Key imaging features distinguishing SMARCB1-deficient sinonasal carcinoma (SDSC) from other entities in the pediatric skull base differential diagnosis.

Entity	Distinguishing imaging features relative to SDSC
Rhabdomyosarcoma	Shares aggressive osseous destruction and diffusion restriction but typically lacks internal calcification and the degree of multicompartment skull base invasion seen in SDSC.
Esthesioneuroblastoma	Arises from the olfactory epithelium with cribriform plate involvement and a dumbbell-shaped intracranial component; usually shows less extensive osseous destruction and lacks the internal calcification pattern of SDSC.
Poorly differentiated chordoma	Central skull base/clival epicenter with osseous destruction, diffusion restriction, and absence of the T2 hyperintensity typical of conventional chordoma; overlaps closely with SDSC and both may show SMARCB1 loss, so distinction ultimately requires immunohistochemical and molecular correlation.
Craniopharyngioma	Suprasellar mass that frequently contains cystic components and rim or nodular calcification; lacks the degree of osseous destruction and diffusion restriction characteristic of SDSC.
Germ cell tumor	Midline location similar to SDSC, but typically contains fat-attenuation or cystic components and lacks the destructive osseous pattern seen in SDSC.
NUT carcinoma	Aggressive midline sinonasal mass that closely mimics SDSC on imaging; reliably distinguished only by immunohistochemistry/molecular testing (NUT rearrangement) rather than imaging features alone.

### Pathology

**Fig. 3:**
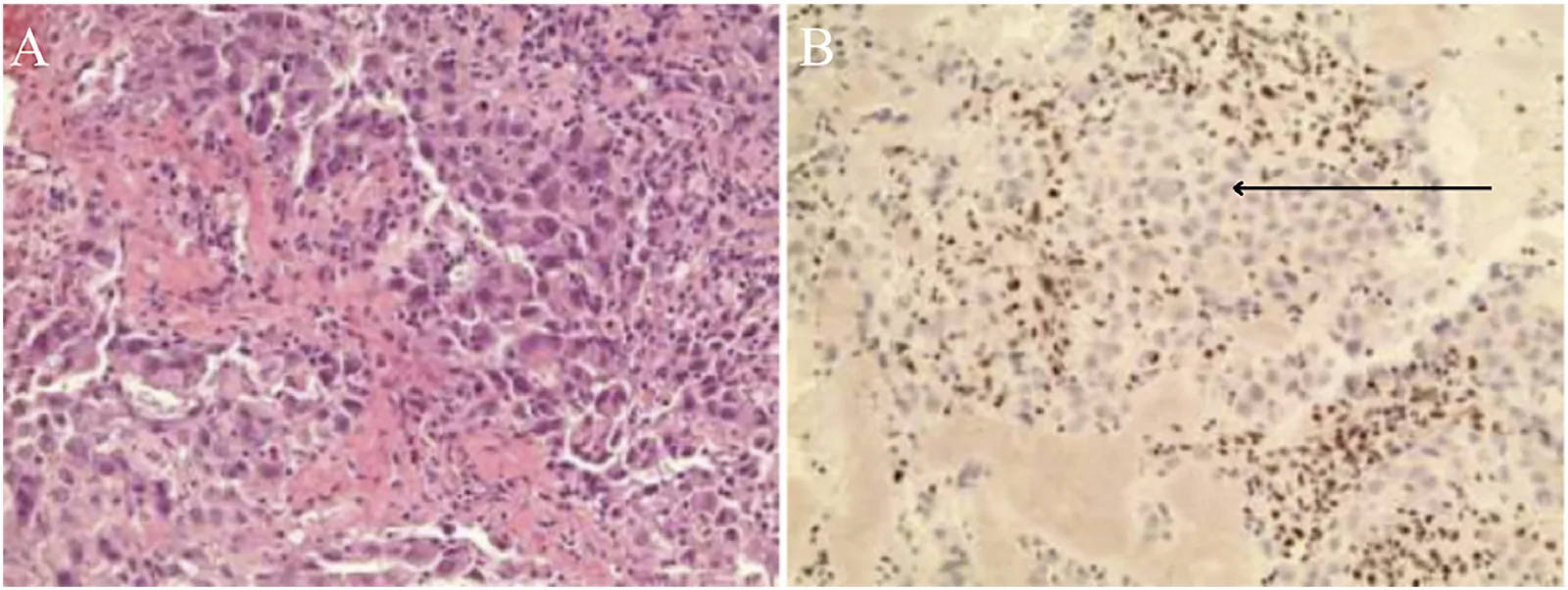
Histopathologic features of SMARCB1-deficient sinonasal carcinoma. (A) Hematoxylin and eosin (H&E) stained section demonstrates a poorly differentiated carcinoma composed of sheets of tumor cells with high nuclear-to-cytoplasmic ratio and hyperchromatic nuclei. (B) Immunohistochemical stain for INI1 (SMARCB1) demonstrates loss of nuclear expression in tumor cells (arrow; nuclei appear pale/unstained), while surrounding inflammatory and endothelial cells retain expected nuclear staining.

Transnasal endoscopic tissue biopsy was performed. Histopathologic analysis demonstrated a poorly differentiated carcinoma with intermixed inflammatory and endothelial cells retaining nuclear expression. Tumor cells demonstrated complete loss of nuclear INI1/SMARCB1 expression on immunohistochemistry, consistent with SMARCB1-deficient sinonasal carcinoma (Fig. 3). Tumor cells were positive for PD-L1 expression. Additional markers that were negative included SALL4, Desmin, Melanoma cocktail, CD45, CD99, CD1a, S100, CK5/6, p63, p40, NUT, OCT4, and EBER ISH. Pan-Keratin was diffusely positive in addition to weakly positive synaptophysin testing (patchy). The tumor retained nuclear expression of BRG1.

### Treatment and imaging follow-up

Following multidisciplinary discussion with pediatric neurosurgery, otolaryngology, and oncology, surgical resection was initially deemed high risk due to extensive cavernous sinus involvement and complete encasement of bilateral optic nerves. The patient underwent two cycles of neoadjuvant chemotherapy with cisplatin and 5-fluorouracil.

Follow-up MRI after two cycles demonstrated a persistent large heterogeneously enhancing skull base mass with scattered areas of diffusion restriction and mild interval decrease in size, measuring approximately 4.4 × 5.8 × 4.4 cm. Surgical resection remained high risk, and the patient received an additional cycle of chemotherapy followed by 6 weeks of radiation therapy to a total dose of 7020 cGy. Subsequent MRI demonstrated overall similar tumor dimensions without substantial interval reduction.

Given tumor PD-L1 positivity, nivolumab therapy was initiated. One month into treatment, the patient developed new facial numbness and worsening headaches. Repeat MRI demonstrated interval increase in tumor size (5.6 × 5.5 × 4.1 cm) with greater involvement of the left orbital apex and further extension into the bilateral pterygopalatine fossae and Meckel’s caves. MR angiography performed at this time demonstrated asymmetric narrowing of the cavernous and supraclinoid segments of the left internal carotid artery secondary to extrinsic compression by the mass.

### Outcome

**Fig. 4:**
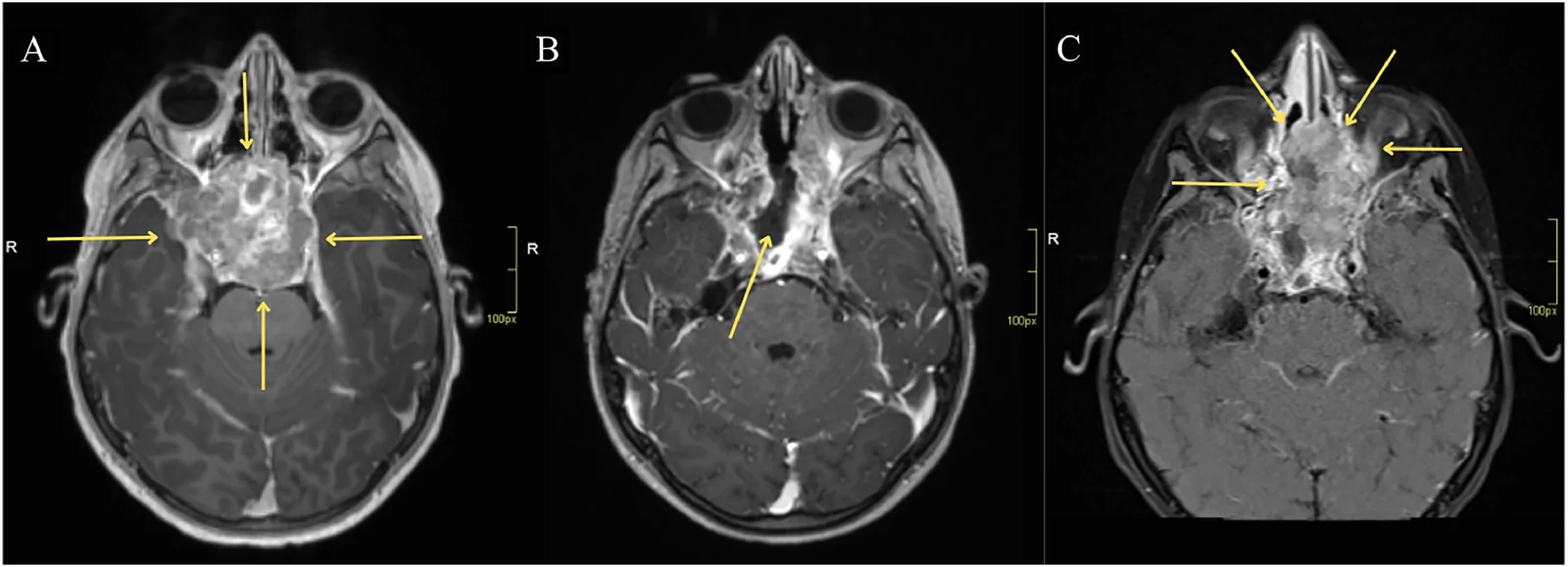
Interval progression of SMARCB1-deficient sinonasal carcinoma despite multimodal treatment. (A) Initial axial contrast-enhanced T1-weighted image demonstrates a large central skull base mass (arrows). (B) Postoperative axial contrast-enhanced T1-weighted image demonstrates a central surgical cavity within the mass following tumor debulking (arrows). (C) Subsequent axial contrast-enhanced T1-weighted image demonstrates interval tumor regrowth with increased anterior extent (arrows).

Due to progressive symptoms and radiologic progression, total resection was again considered but deemed infeasible given extensive cavernous sinus and bilateral optic nerve involvement. Subtotal endoscopic resection via a transnasal transsphenoidal approach was performed. Postoperative imaging demonstrated substantial residual disease involving the central skull base, cavernous sinuses, and orbital apices. Repeat MRI demonstrated further disease progression despite multimodal therapy and surgical debulking (Fig. 4).

Shortly thereafter, the patient developed left cervical lymphadenopathy. Biopsy confirmed metastatic disease. Repeat PET/CT imaging demonstrated further disease progression with increased FDG activity in the bilateral anterior arches of C1 and bilateral level IIa lymph nodes. Multiple FDG nodules in the bilateral lungs were noted. Additional FDG nodules were visualized in the mediastinal, mesenteric, and bilateral hilar lymph nodes.

**Fig. 5:**
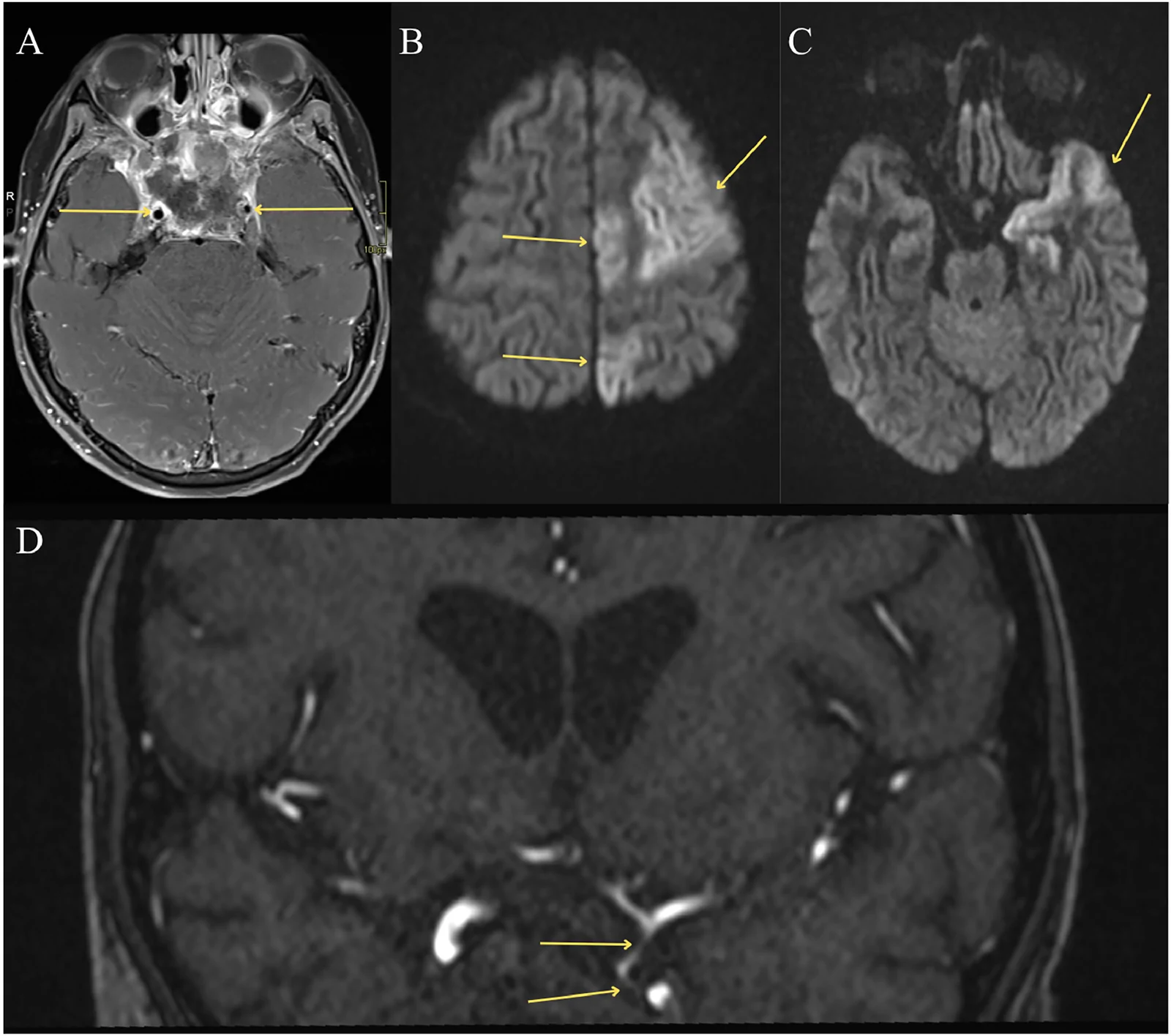
Vascular complications of SMARCB1-deficient sinonasal carcinoma. (A) Axial contrast-enhanced T1-weighted image demonstrates tumor encasing the bilateral internal carotid arteries (arrows), with asymmetric narrowing of the left internal carotid artery. (B) Diffusion-weighted imaging demonstrates acute infarction within the left cerebral hemisphere, predominantly involving the MCA territory with additional ACA watershed infarcts (arrows). (C) Diffusion-weighted imaging at a more inferior level demonstrates additional infarction involving the left anterior temporal lobe (arrow), consistent with an MCA infarct. (D) Subsequent MR angiography demonstrates severe narrowing to near-complete occlusion of the left internal carotid artery (arrows).

Approximately 18 months after diagnosis, the patient developed acute neurologic decline with stroke-like symptoms. Imaging demonstrated focal stenosis of the left paraclinoid internal carotid artery and multiple left middle cerebral artery infarcts representing a complication of progressive vascular encasement by the tumor (Fig. 5). The patient died from these complications shortly thereafter. Concise timeline of initial presentation, diagnosis, treatment, and outcomes are outlined in Table 2.

## Clinical timeline

**Table 2:** Timeline of diagnosis, treatment, and disease progression.

Time point	Event
Presentation	8-year-old boy presents with new-onset right vision loss and right afferent pupillary defect.
Diagnostic workup	Contrast-enhanced MRI, CT, and PET demonstrate a large, heterogeneously enhancing central skull base mass with sheet-like calcification and multicompartment extension; endoscopic biopsy confirms SMARCB1-deficient sinonasal carcinoma.
Treatment initiation	Two cycles of neoadjuvant chemotherapy (cisplatin/5-fluorouracil); surgical resection deferred due to cavernous sinus and bilateral optic nerve encasement.
After 2 cycles chemotherapy	Follow-up MRI shows persistent mass with mild interval decrease in size; a third chemotherapy cycle is given, followed by 6 weeks of radiation therapy (7020 cGy).
After chemoradiation	Subsequent MRI shows overall similar tumor dimensions without substantial reduction; nivolumab (anti-PD-1) initiated given tumor PD-L1 positivity.
1 month after starting nivolumab	New facial numbness and worsening headaches; MRI shows interval tumor growth with new left orbital apex and Meckel’s cave involvement and left ICA narrowing on MRA.
Surgical intervention	Subtotal endoscopic transnasal transsphenoidal resection performed; postoperative imaging shows substantial residual disease, and repeat MRI shows further progression.
Metastatic progression	Development of left cervical lymphadenopathy with biopsy-confirmed metastasis; PET/CT shows further progression with nodal, pulmonary, mediastinal, and mesenteric involvement.
∼18 months after diagnosis	Acute neurologic decline with stroke-like symptoms; imaging demonstrates left paraclinoid ICA stenosis and multiple left MCA infarcts from progressive vascular encasement. The patient died shortly thereafter.

## Discussion

SMARCB1-deficient sinonasal carcinoma (SDSC) in this pediatric patient demonstrates many of the aggressive imaging characteristics described in adults. In adult series, SDSC is characterized as a high-grade sinonasal malignancy frequently presenting with advanced locoregional disease [11]. Previously reported imaging findings include heterogeneous enhancement, diffusion restriction consistent with hypercellularity, and osseous destruction [5], all of which were observed in this case. Shatzkes et al. reported calcifications in approximately half of adult cases, with some demonstrating a “hair-on-end” periosteal pattern [5]. In contrast, our case demonstrated a more sheet-like internal calcification pattern on CT. While the significance of this difference remains to be established, it suggests that imaging features in pediatric cases may not fully mirror those reported in adults and therefore warrant further investigation as additional pediatric cases are described. Given the limited number of reported pediatric cases, this sheet-like calcification pattern should currently be regarded as a potentially useful but anecdotal imaging observation rather than an established diagnostic feature; larger pediatric cohorts will be needed to determine whether it is reproducible and diagnostically specific for SDSC. The extent of local invasion at presentation reflects the high-grade nature of this lesion and underscores the importance of early radiologic recognition.

Although sinonasal malignancies are rare in children, the differential diagnosis of an aggressive pediatric skull base mass is broad and includes entities such as rhabdomyosarcoma, esthesioneuroblastoma, poorly differentiated chordoma, craniopharyngioma, germ cell tumor, and NUT carcinoma. This case highlights that SDSC should be considered in this differential, particularly when imaging demonstrates multicompartment skull base invasion, diffusion restriction, and internal calcification. Because SDSC has not historically been considered in the pediatric population, immunohistochemical evaluation for SMARCB1/INI1 loss may have been omitted, raising the possibility that prior pediatric cases have been misclassified. Recognition of this constellation of imaging findings may prompt appropriate immunohistochemical workup and facilitate an accurate diagnosis.

Loss of SMARCB1 function is classically associated with atypical teratoid/rhabdoid tumor and malignant rhabdoid tumor, and germline SMARCB1 mutations have been identified in as many as 35% of rhabdoid tumors [12]. Although a direct germline association between SMARCB1-deficient sinonasal carcinoma and rhabdoid tumor predisposition has not been definitively established, identification of SMARCB1 loss in a pediatric patient warrants consideration of genetic evaluation given the potential implications for surveillance for additional SMARCB1-associated tumors and counseling regarding familial risk. Germline testing was not performed in this case; however, clinicians encountering SMARCB1 loss in pediatric patients should consider referral for genetic counseling.

SDSC is associated with a poor prognosis, with previously reported median survival of approximately 22 months in adults [11]. The rarity of this entity has limited the development of standardized treatment protocols, with fewer than 200 cases reported in the literature as of 2022^5^. Documented responses to platinum-based chemotherapy, anti-PD-1/PD-L1 immunotherapy, and emerging EZH2 inhibitor therapies provide a basis for therapeutic decision-making, though no consensus protocol exists [13,14]. In this case, multimodal therapy including chemotherapy, radiation, immunotherapy, and subtotal resection was ultimately unable to control disease progression, consistent with the aggressive nature of the lesion reported in adult series. Serial imaging played a central role in monitoring treatment response, detecting disease progression, and guiding therapeutic decision making throughout the patient's clinical course.

## Key imaging features suggestive of SMARCB1-deficient sinonasal carcinoma

• Aggressive central skull base mass with multicompartment extension.
• Heterogeneous enhancement with diffusion restriction.
• Internal “sheet-like” or “hair-on-end” calcification.
• Early cavernous sinus and orbital apex involvement.
• Encasement or narrowing of adjacent neurovascular structures.

## Limitations

This report is limited by its single-case design, which precludes definitive conclusions regarding the generalizability of imaging findings. Though the imaging findings presented in this report represent unique findings when compared to imaging features observed in adults, a single case does not provide adequate evidence for generalized diagnostic specificity.

## Conclusion

SMARCB1-deficient sinonasal carcinoma is a recently characterized entity that has been described predominantly in adults, with only rare reports in adolescents. This case demonstrates that the tumor can also occur in children as young as 8 years of age and may present with advanced multicompartment skull base involvement at the time of diagnosis. Given the overall rarity of sinonasal malignancies in pediatric patients, this entity may not be initially considered, and without appropriate immunohistochemical evaluation, cases could potentially be misclassified.

Recognition of its aggressive imaging appearance, including multicompartment skull base invasion, diffusion restriction, and internal calcification, may provide an early opportunity to suggest SMARCB1/INI1 deficiency. When this constellation of findings is present in a pediatric patient, inclusion of INI1/SMARCB1 immunohistochemistry in the pathologic workup is essential for accurate classification. Increased awareness of this entity in the pediatric population may improve diagnostic precision and support earlier multidisciplinary management of this aggressive malignancy.

## Patient consent

Written, informed consent was obtained from the patient’s representative prior to the publication of this case report, including consent for the use of accompanying clinical imaging, data, or other potentially identifiable information. Patients were informed of the nature of the publication, potential audience, and their right to withdraw consent without consequence to their care.
